# PineElm_SSRdb: a microsatellite marker database identified from genomic, chloroplast, mitochondrial and EST sequences of pineapple (*Ananas comosus* (L.) Merrill)

**DOI:** 10.1186/s41065-016-0019-8

**Published:** 2016-11-24

**Authors:** Sakshi Chaudhary, Bharat Kumar Mishra, Thiruvettai Vivek, Santoshkumar Magadum, Jeshima Khan Yasin

**Affiliations:** Division of Genomic Resources, ICAR- National Bureau of Plant Genomic Resources, PUSA campus, 110012 New Delhi, India

**Keywords:** Ananas, Genome wide marker analysis, Organelle, Pineapple, Simple sequence repeats

## Abstract

**Background:**

Simple Sequence Repeats or microsatellites are resourceful molecular genetic markers. There are only few reports of SSR identification and development in pineapple. Complete genome sequence of pineapple available in the public domain can be used to develop numerous novel SSRs. Therefore, an attempt was made to identify SSRs from genomic, chloroplast, mitochondrial and EST sequences of pineapple which will help in deciphering genetic makeup of its germplasm resources.

**Results:**

A total of 359511 SSRs were identified in pineapple (356385 from genome sequence, 45 from chloroplast sequence, 249 in mitochondrial sequence and 2832 from EST sequences). The list of EST-SSR markers and their details are available in the database.

**Conclusions:**

PineElm_SSRdb is an open source database available for non-commercial academic purpose at http://app.bioelm.com/ with a mapping tool which can develop circular maps of selected marker set. This database will be of immense use to breeders, researchers and graduates working on *Ananas* spp. and to others working on cross-species transferability of markers, investigating diversity, mapping and DNA fingerprinting.

**Electronic supplementary material:**

The online version of this article (doi:10.1186/s41065-016-0019-8) contains supplementary material, which is available to authorized users.

## Background

The extremely surprising flavour and fragrance of pineapple (*Ananas comosus* L*.*) delighted mankind at that time of its discovery by Christopher Columbus and even today. Pineapple, a perennial monocot plant belongs to Bromeliales order, Bromelioideae subfamily and Bromeliaceae family. Pineapple is a tropical plant native to South America, domesticated more than 6000 years ago [1]. At the end of the sixteenth century, pineapple had become pantropical and is the third most economically important tropical fruit crop after banana and mango. Pineapple has become industrial crop during 20^th^ century [2,3]. In addition to fresh fruit consumption, pineapple is used for canned slices, juice and juice concentrate, extraction of bromelain (a meat-tenderizing enzyme), high-quality fibre, animal feed and medicines [2]. At present, gross production value of pineapple is approaching $9 billion due to its cultivation on 1.02 million hectares of land in over 80 countries and annual production of 24.8 million metric tonnes of fruit [4]. Wild varieties of pineapple are self-compatible, whereas cultivated pineapple, *A. comosus* (L.) Merr., is self-incompatible [5], which provides an opportunity to scrutinize the molecular basis of self-incompatibility in monocots.

Over the last few decades, a wide range of molecular markers have been developed and used in crop improvement as molecular markers are helpful in assessing germplasm diversity, testing of hybridity, trait mapping, marker assisted selection etc. [6]. Among all the markers till date, Simple Sequence Repeats (SSRs) are the most ideal, powerful and reliable markers for molecular plant breeding applications because of their high abundance, co-dominant inheritance and multiple alleles [7]. In addition, BES-SSR markers serve a useful resource for integrating genetic and physical maps [8,9].

SSRs consists of 2–7 base pair tandem repeat motifs of mono-, di-, tri-, tetra and penta-nucleotides (A, T, AT, GA, AGG, AAAG etc.) with different lengths of repeat motifs. These repeats are extensively distributed throughout plants and animal genomes. A high level of genetic variation is observed between and within species due to differences in the number of tandem repeating units at a locus which produces a highly polymorphic banding pattern [10] and is detected by the Polymerase Chain Reaction (PCR) using locus specific flanking primers [11]. Molecular markers are widely recognized as a tool in generating linkage maps [12] as they define specific locations in the genome unambiguously [13,14].

There are few valuable software and tools available for SSRs identification and *in-silico* marker development. Important sources for SSR identification are with benefits from the advanced next generation sequencing technology such as TROLL [15], MISA [16], SciRoko [17], SSR Locator [18] and GMATo [19]. MISA is the most common tool used for SSR identification. Generation of SSR markers have been exhaustive due to the time-consumption, expensive process for generation of genomic libraries and sequencing of large number of clones later to find the SSR-containing DNA regions [20] and labour-intensive. To expedite this task, the traditional methods of SSR markers generation from genomic libraries [21] have been recouped briskly by *in-silico* mining of SSRs from DNA sequences available in biological databases [22,23] and from expressed sequence tags (ESTs) that represent only the coding region of the genome [24–26].

## Methods

### Retrieval of genome sequences

The complete genome sequence of pineapple (*Ananas comosus* (L.) Merrill) was retrieved from the CoGe Genome (Genome ID- 25734) page (https://genomevolution.org/coge/GenomeInfo.pl?gid=25734) in FASTA format. The chloroplast genome (Genome ID- 25280) and mitochondrial genome (Genome ID- 25281) of pineapple were also downloaded from CoGe Genome info respectively (https://genomevolution.org/coge/GenomeInfo.pl?gid=25280&81) in FASTA format. Total 5978 EST sequences of pineapple were downloaded from NCBI http://www.ncbi.nlm.nih.gov/nucest/?term=ananas+comosus in FASTA format.

### SSRs identification

MISA tool allows the identification and localization of perfect microsatellite as well as compound microsatellite which are interrupted by a certain number of bases. MISA uses Perl script for SSRs analysis. It requires a set of sequences in FASTA format and a parameter file that defines unit size and minimum repeat number of each SSR. MISA is available at http://pgrc.ipk-gatersleben.de/misa/. MISA tool provides two result files; misa file and statistical file. MISA file provides the information about SSR repeat types like simple, interrupted or compound, size of SSR and SSR position in genome sequence. Statistical file contains the statistical information like the frequency chart of SSR motif and distribution of SSR to differently repeat type classes. Classification of SSRs was done manually on the basis of their presence in coding region and non-coding region of the genome sequences.

### Database development

An open, non-commercial database PineElm_SSRdb is designed for educational purpose. PineElm_SSRdb is available at http://app.bioelm.com/.

## Results and discussion

**Fig. 1:**
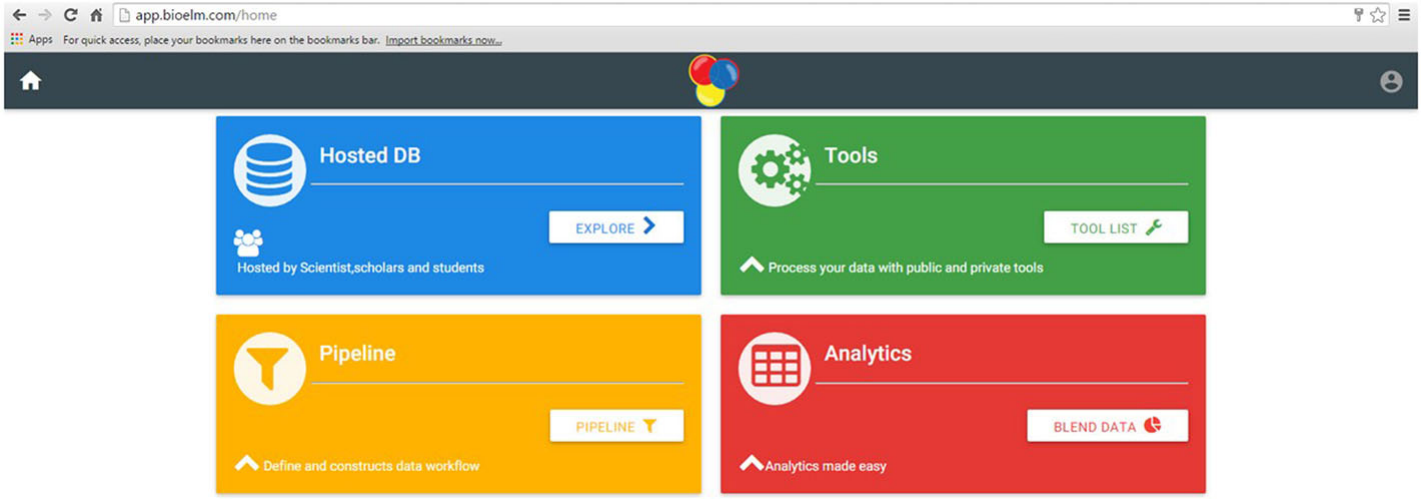
Homepage of the web app

**Fig. 2:**
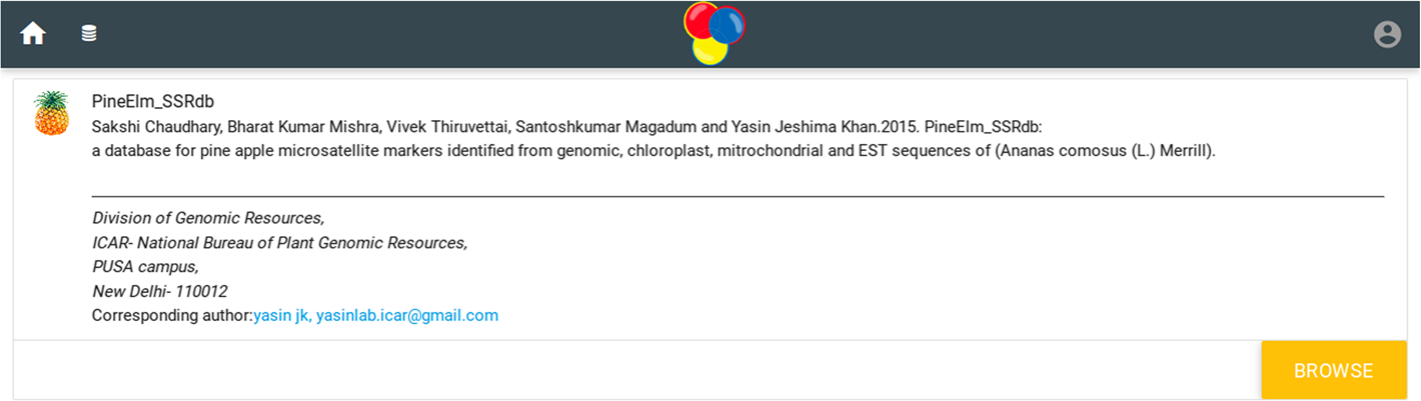
Main page of the database

**Fig. 3:**
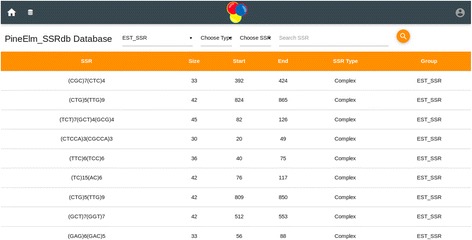
EST-SSRs list

**Fig. 4:**
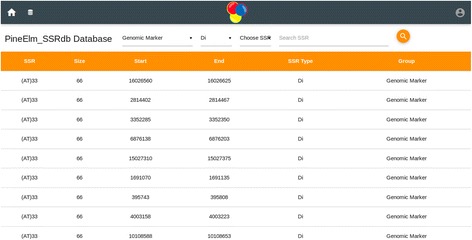
Genomic SSR marker list

The home page of the database provides the complete access of the database (Figs. 1 and 2). Genomic sequence of *Ananas comosus* (L.) Merrill is available as 3133 scaffolds of 381,905,120 bp length. Of these scaffolds only 2726 contain SSRs. From genomic sequence 356385 SSRs were identified. Two thousand four hundred eighty-six sequences contain more than one SSR and 19086 compound SSRs are also exists. From NCBI 5978 EST sequences of 4,294,909 bp were downloaded of which 1886 sequences yielded 2832 SSRs (Fig. 3). Of these 1886 sequences 638 were found to contain more than one SSR region and 83 with compound repeat regions. The access to the database can be obtained from http://app.bioelm.com/ by creating user account. This will allow the users to generate images and search results. Genomic markers can be viewed as listed in Fig. 4 and as a circular map of the selected markers using incorporated tools (Additional file 1).

**Fig. 5:**
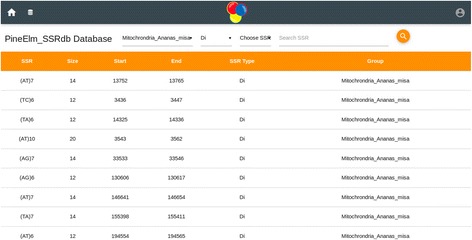
Mitochondrial SSR marker list

There is only one chloroplast genome sequence of 159,600 bp available for *Ananas comosus* (L.) Merrill in which there are 45 SSRs exists and only one complex repeat (Additional file 2). There are 13 sequences of 881,399 bp *Ananas comosus* (L.) Merrill mitochondrial genome yielded 249 SSRs of which 13 sequences contain more than one SSR and 8 SSRs were found in compound formation (Fig. 5 and Additional file 3). The EST-SSR statistics were represented in Additional file 4. SSRs identified from chloroplast and mitochondrial sequences are highly specific and unique to pineapple. These SSRs are not present in any other NCBI database and also not present in the nuclei genome of pineapple.

Databases support instant availability of curated data for individual users in facilitating further effective use of the generated data. In that path, we have developed a database to support pineapple breeders to effectively use SSR markers in their breeding program. These SSR markers or microsatellites are of 1–6 nucleotide tandem repeated motifs present in all prokaryotic and eukaryotic genomes [27]. Amid different classes of available molecular markers, SSR markers are effective for a variety of applications in plant genetics and breeding [28, 29].

Although being a commercially important plant, only few studies for SSR development were available for Pineapple. Wohrmann and Weising [30], identified 696 EST-SSR markers in pineapple; Feng et al. [31] developed genomic and EST-SSR library to identify 94 and 1110 SSRs loci respectively. Complete Pineapple genome [4] opens new direction to focus our research towards pineapple. In addition, bioinformatics tools also add-on prevailing methods by automating the assignment of SSR identification from existing DNA sequences. A recent study reported 320,207 SSRs in genomic and ESTs sequences of pineapple [32]. Whereas, we have identified 356385 SSRs from genomic sequences of pineapple which may play a major role in diversity analysis of genetic stocks. Diversity analyses of pineapple genetic stocks were reported earlier with few markers which were insufficient in to distinguish them. Developing fingerprints of cultivars may be required to protect the breeders right. Genome wide identification of markers can serve this purpose as SSR markers have been handy for integration the physical, genetic and sequence-based physical maps in plant species, and concurrently equipped breeders and geneticists with an effective tool to bridge phenotypic and genotypic variation. SSR markers have been handy for integration the physical, genetic and sequence-based physical maps in plant species, and concurrently equipped breeders and geneticists with an effective tool to bridge phenotypic and genotypic variation [33].

**Fig. 6:**
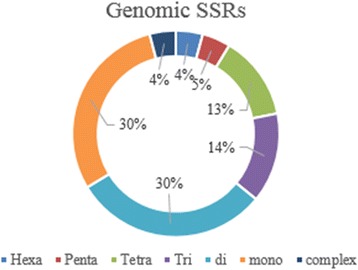
Distribution of Genomic SSR markers

**Fig. 7:**
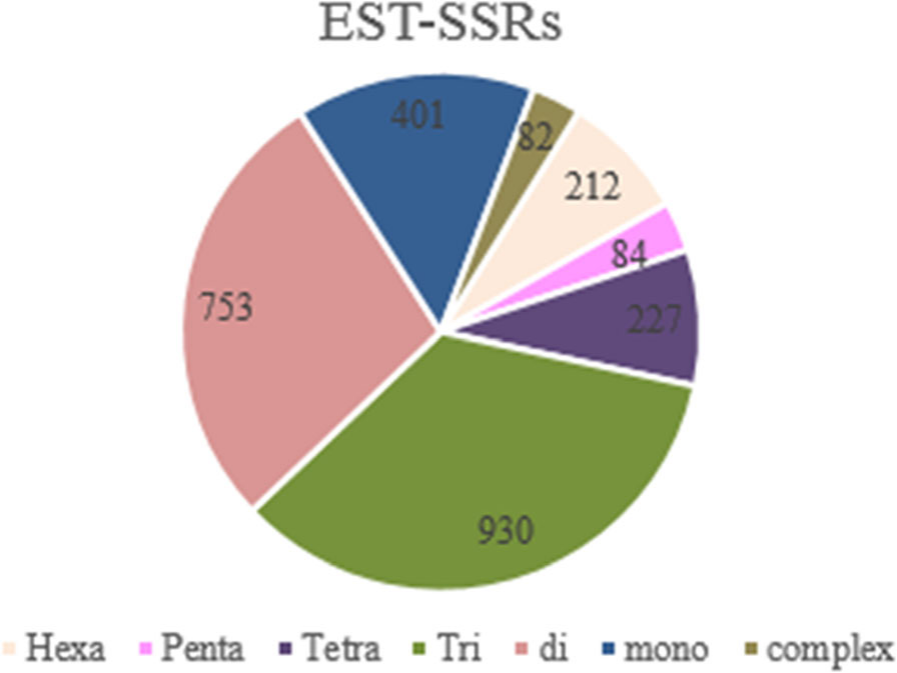
Distribution of EST- SSR markers

**Fig. 8:**
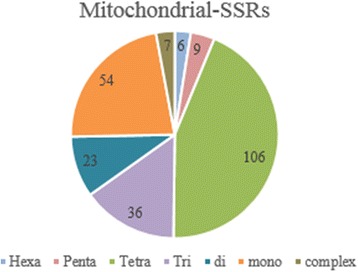
Distribution of mitochondrial SSR markers

SSR markers were classified based on number of repeats (Figs. 6, 7 and 8). The proportion of mono and di repeats are likely to be equal for genomic SSRs contributing to the total 60% of genomic SSR markers. Likewise, hexa and complex repeats are approximately equal at 4% of genomic SSRs (Fig. 6). Recent studies of plant markers are more focused towards gene-specific markers rather than arbitrary DNA markers, and microsatellite markers are of great significance in identification of genes and QTLs [34]. EST-SSRs are highly efficient in differentiating genotypes differing for a specific trait. We have identified 2832 SSR markers from the validated EST sequences, where tri repeats followed by di repeats contributes more to the markers (Fig. 7). Tetra and mono repeats contribute maximum number of mitochondrial – SSRs (Fig. 8). In all sets of data analysed, we could find least number of complex repeats and only one complex SSR exists in chloroplast genome of pineapple.

Molecular basis of polymorphism and their distribution across the genome is quite different for SNP and SSR markers. Both SSR and SNP are neutral, multi-allelic and co-dominant markers. SSR marker in genetic diversity analyses have been a powerful, handy, cost effective tool and can reveal the amplicon size polymorphism as they vary in sequence, whereas SNP haplotypes vary within a sequence. SNP markers display population structure better with bigger population whereas, for diversity analyses, SSR unveils better grouping of accessions even at trait level. Further, it has been demonstrated that haplotypes at combinations of SSR loci may be very powerful in detecting association of QTLs (Quantitative Trait Loci) in their proximity [35]. Henceforth, the utility of SSR/SNP marker in crop improvement will depend on the quality of information required with respect to parameters for genetic diversity and population structure. Overall, to assess genetic relatedness, SSR markers are more informative and highly effective [36].

## Conclusion

The main outcome of this study; identified SSRs markers in genomic, chloroplast, mitochondrial and EST sequences of Pineapple will be of immense use to breeders and molecular biologists to assess marker frequency and distribution in both coding and non-coding regions, to study transferability across genera and to carry out phylogenetic analysis based on SSRs. PineElm_SSRdb is an open source database developed for easy handling and availability for the scientific community.

## Additional files

Additional file 1: Genomic SSR markers of Ananas comosus (L.) Merrill. (TXT 5249 kb)
Additional file 2: Chloroplast SSR markers of Ananas comosus (L.) Merrill. (TXT 1 kb)
Additional file 3: Mitochondrial SSR markers of Ananas comosus (L.) Merrill. (TXT 7 kb)
Additional file 4: EST SSR markers of Ananas comosus (L.) Merrill. (TXT 42 kb)
